# Factors influencing social and health outcomes after motor vehicle crash injury: an inception cohort study protocol

**DOI:** 10.1186/1471-2458-14-199

**Published:** 2014-02-25

**Authors:** Jagnoor Jagnoor, Fiona Blyth, Belinda Gabbe, Sarah Derrett, Soufiane Boufous, Michael Dinh, Robert Day, Gregory Button, Mark Gillett, Tony Joseph, Michael Nicholas, Rebecca Ivers, Chris G Maher, Simon Willcock, Justin Kenardy, Alex Collie, Ian D Cameron

**Affiliations:** 1 Rehabilitation Studies Unit, Sydney Medical School Northern, Level 13, Kolling building, St Leonards, NSW, Australia; 2 E25, Royal North Shore Hospital, The University of Sydney, Sydney, Australia; 3 The George Institute for Global Health, PO Box M201 Missenden Road, Sydney, Australia; 4 C22, Concord Clinical School, The University of Sydney, Sydney, Australia; 5 Epidemiology & Preventative Medicine, Monash Medical School Building, The Alfred Hospital, Melbourne, Victoria, Australia; 6 Preventive & Social Medicine, Injury Prevention Research Unit (IPRU), PO Box 56, Dunedin, New Zealand; 7 Transport and Road Safety (TARS), University of New South Wales, Research Level 1, West Wing, Kensington, Australia; 8 C39, Royal Prince Alfred Hospital, The University of Sydney, Sydney, Australia; 9 Orange Base Hospital, PO Box 2052, Orange, NSW, Australia; 10 The University of Sydney, 37A Booth Street, Balmain, NSW, Australia; 11 CONROD, University of Queensland, Level 1, Edith Cavell Building, Herston, Australia; 12 Institute of Safety, Compensation and Recovery Research, Level 11, 499 St Kilda Road, Melbourne, Victoria, Australia

## Abstract

**Background:**

There is growing evidence that health and social outcomes following motor vehicle crash injury are related to cognitive and emotional responses of the injured individual, as well as relationships between the injured individual and the compensation systems with which they interact. As most of this evidence comes from other states in Australia or overseas, investigation is therefore warranted to identify the key determinants of health and social outcomes following injury in the context of the New South Wales motor accident insurance scheme.

**Methods/Design:**

In this inception cohort study, 2400 participants, aged 17 years or more, injured in a motor vehicle crash in New South Wales will be identified though hospital emergency departments, general and physiotherapy practitioners, police records and a government insurance regulator database. Participants will be initially contacted through mail. Baseline interviews will be conducted by telephone within 28 days of the injury and participants will be followed up with interviews at 6, 12 and 24 months post-injury. Health insurance and pharmaceutical prescription data will also be collected.

**Discussion:**

The study results will report short and long term health and social outcomes in the study sample. Identification of factors associated with health and social outcomes following injury, including related compensation factors will provide evidence for improved service delivery, post-injury management, and inform policy development and reforms.

**Trial registration:**

Australia New Zealand Clinical trial registry identification number - ACTRN12613000889752. Available at: ANZCTR Registered FISH Study.

## Background

Worldwide, road traffic injuries are ranked twelfth in terms of contribution to Disability Adjusted Life Years [1]. Whilst 90% of the road traffic injury burden falls on low or middle income countries [1,2], it also has major personal and societal implications in high income countries such as Australia [3].

The economic loss associated with road traffic injuries in Australia is equivalent to nearly 3% of the national Gross Domestic Product. The 2009 report by The Australian Bureau of Infrastructure, Transport and Regional Economics estimated a total cost, in Australian Dollars (AUD), of $2.8 billion, associated with road traffic injuries in the nation’s most populous state of New South Wales (NSW) [4]. Nearly 70% of this cost was associated with human factors such as disability, costs of medical services, lost productivity and insurance administration. Clearly, understanding factors associated with the human costs are a priority for reducing the socio-economic burden of injury arising from road traffic injuries [4].

There is growing evidence that health and social outcomes following road traffic injuries are related to the cognitive and psychosocial responses of the injured person [5], the interaction between the injured individual and the systems they experience following an injury [6-8]. This is in addition to the specific physiological and anatomical effects of the injury. Studies have also suggested that pre-injury socio-demographic and health characteristics are associated with adverse functional and disability outcomes after injury, independent of the anatomical nature and severity of injury [9,10].

Many studies have investigated recovery after road traffic injuries; however the focus of the research has generally been on particular types of injury such as orthopaedic injury or whiplash associated disorders [11-13]. In a time series study, it has been shown that health outcomes of people with whiplash injury improved after legislative change. The changes included removal of financial compensation for "pain and suffering" for whiplash, introducing clinical practice guidelines for its treatment; and change in regulations to permit earlier acceptance of compensation claims, and earlier access to treatment [14].

The ESPARR cohort from France reported that only 45% of the participants with mild-to-moderate injury in road traffic crash reported to have fully recovered from the injury [15]. In Victoria, Australia, cohort studies following up orthopaedic and trauma patients have reported worse short term and long term health, vocational and functional outcomes, among people claiming compensation through Transport Accident Commission (TAC) [11,16]. Similar findings have also been reported in context of work injuries, with injured people receiving compensation having worse health outcomes and a slower recovery than people with similar injuries not receiving compensation [17,18]. In another study in Victoria, 8% of participants reported loss of earnings and a median of 33 days of work disability for injuries that did not require any hospital stay [19].

As insurance and compensation systems vary by jurisdiction, and there has been little research identifying key factors related to recovery in NSW. The Motor Accident Authority in NSW, has identified several limitations in the current compulsory scheme, for example the scheme need to establish fault means the NSW CTP Scheme is essentially adversarial, delayed payments, high cost and disputes [20]. Hence, further investigation is warranted to confirm the key determinants of health and social outcomes in a broader cohort of people following road traffic injury in NSW, and in particular to identify the specific features of the compensation process that might facilitate or impede recovery.

This study takes a broad view. It investigates the potential factors that may influence health and social recovery, including pre- injury socio-demographic and health characteristics, injury characteristics, utilisation of health services, and compensation factors such as claims process, treatment, liability and fault, and legal representation.

### Objectives

The specific objectives of the study in context of the New South Wales, compulsory third party scheme are as follows:

1. To determine whether particular prognostic factors, including those related to health, socio-demographic and economic status, are predictors of outcome following injury.

2. To determine whether particular factors linked to compensation are predictors of outcome.

## Methods/Design

### Settings

NSW is the most populous state of Australia with an estimated population of 7.29 million that is 34.5% of the population of Australia. Over 73% of the state’s population lives in cities. There were over 5,876,000 vehicles registered and 5,655,000 licensed drivers in NSW in 2013. Nearly 42,000 crashes are recorded every year with over half of the crashes leading to casualties [10].

Compensation from the third party insurance scheme (also called Compulsory Third Party insurance or CTP), is available for persons killed or injured as a result of a motor vehicle crash. The scheme does not cover the at fault driver; although there are exceptions for people with very severe injuries (from 1 October 2007 for adults). It provides benefits for injured persons that include medical treatment, rehabilitation expenses, compensation for lost earnings, and for other accident-related expenses. The scheme is designed to support early treatment and recovery. In the 2011–2012 financial year, approximately 14,000 claims were made and payments to injured people were approximately $AUD 1,290 million [21].

### Design

The study will utilise an inception cohort design. Recruitment will be conducted through metropolitan and rural hospital emergency departments, general practitioners, physiotherapy clinics, police crash records and the claims database of a government insurance regulator (the NSW Motor Accidents Authority). Minimal information to establish eligibility will be collected from study sites/data sources. Detailed inclusion and exclusion criteria are reported in Table 1.

**Table 1:** Inclusion and exclusion criteria for the FISH study

**Inclusion criteria**	
	Injury due to motor vehicle crash diagnosed by a medical practitioner, or registered health practitioner, within 28 days of the crash
	Injury due to crash involving a motorised vehicle on land (public/private road/driveway/parking space or private/public land) in NSW
	Injured person is a driver, rider, passenger, pillion passenger, pedestrian (person travelling on foot or operating toy vehicle, pedal car, barrow, billycart, non-motorised wheelchair or skateboard) or cyclist
	Adults aged 17 years or over
	Potential participants from a Non-English Speaking Background (NESB) are included if they are able to answer the interview questions
	Potential participant must be residents of NSW, with a valid Medicare number
**Exclusion criteria**	
	Injury due to crash involving types of land transport other than motorised vehicle such as trains and light rail that are not covered by the CTP
	Dementia or significant pre-existing cognitive impairment affecting ability to consent
	Severe injury, which includes severe traumatic brain injury, spinal cord injury, extensive burns or multiple amputations as these injuries are covered defined by the NSW Lifetime Care and Support Scheme
	Isolated, superficial soft tissue injuries such as bruises, abrasions or cuts were also excluded as these very minor injuries are not covered by the CTP
	Injury occurring as a result of intentional self-harm
	Death of an immediate family member in the motor vehicle crash

### Participant identification and sampling

Participants will be recruited from the following sites:

*Hospital Emergency Departments:* Data will be collected at two major Sydney metropolitan region hospitals (Royal Prince Alfred and Royal North Shore) recruiting 600 participants from each hospital. In addition, approximately 400 participants will be identified from three emergency departments in rural NSW (Orange, Dubbo and Bathurst Health Services). Research nurses at each site will screen the “First Net” emergency department data base to identify potential participants.

*General practitioners:* We aim to recruit 200 participants through General Practitioners at two urban primary healthcare networks: the North Shore Medicare Local and Inner West Medicare Local. Medicare Local services are geographically-defined organisations that coordinate the provision of community based healthcare in their region. General practitioners will be reimbursed for their time for assisting in identifying participants for the study.

*Physiotherapists:*Twenty physiotherapy practices will be approached by study staff in the Sydney region to identify potential participants for the study. Recruitment of approximately 200 participants is planned from physiotherapy practices. Physiotherapists will be reimbursed for their time for assisting in identifying participants for the study.

The screening data from study sites will be entered on a secure online platform, Research electronic data capture (REDCap) [5].

*Police Crash Data source:* Police data will be accessed to identify 150 participants with non-catastrophic injury from rural New South Wales.

*NSW Motor Accidents Authority Registry:* Approximately 200 potential participants will be identified on a monthly basis from the Claims Advisory database and Personal Injury Registry. These are claims databases maintained by the government motor accident insurance regulator.

### Recruitment and consent

From the study sites, data for potential participants based on eligibility criteria will be entered on a secure online platform, Research electronic data capture (REDCap) [5]. Once screened, potential participants will be sent a letter which details the purpose of the study, what is involved and inviting them to participate in the study. Participants can opt-out of the study via phone or through email. Participants who do not opt-out, within one-week of the letter mail-out, will be contacted by trained interviewers. Interviewers obtain informed consent by telephone and conduct the baseline structured interview. Participants will be asked to consent to their data being accessed from Medicare (the Australian universal health insurance scheme) and from the Pharmaceutical Benefit Scheme, and to being contacted for future follow-up interviews [22]. The interviews will be conducted using Computer Aided Telephone Interview (CATI) by trained interviewers. Follow-up interviews will be conducted by telephone or through e-mail at 6, 12 and 24 months. It is anticipated that 70 to 85% of the cohort will be retained at 24 months [10].

Health and social recovery outcome data and variables that potentially influence these outcomes will be collected though a telephone interview using multiple standardised measures, as reported in Table 2. The focus is on key variables known to be associated with health outcomes. The duration of baseline telephone interview will be no longer than 45 minutes and follow –up interviews will be of approximately 10–15 minutes.

**Table 2:** Data collected at baseline and follow-up for the FISH study

**Baseline**	
Socio-demographic	Age, sex, place of birth, primary language, education, marital status
Employment	Employment status, occupation, income
Health	BMI ( height and weight), history of chronic illness
Health related quality of life	Likert scale for overall health, EQ5D3L [23] and SF12 [24] prior to crash and at baseline, Global Perceived Effect (GPE) [25].
Lifestyle habits	Smoking status and alcohol consumption
Pain	Numeric Rating Scale (NRS) [26], Pain Catastrophising Scale (PCS) [27], Orebro Musculoskeletal Pain Question (OMPQ, short form) [27].
Disability and functioning	History of any disability, World Health Organisation Disability Assessment Schedule II (12-item version) (WHODAS II) [28].
Psychological factors	Perceived threat to life and disability, Depression, Anxiety and Stress Scale (DASS21) [29] and Impact of Events Scale (IES-R) [30], expectation to recover using OMPQ.
Health Care Utilisation	Visit to health care professionals 3 months prior to crash, history of health care utilization in 12 months prior to crash through Medicare data linkage
Injury	Type of injury and associated hospitalisation
Crash related factors	Role, perceived danger of death and disability
Work	Return to work, modified duties, hours
Social life	Satisfaction with social relationships
6*, 12 and 24 months follow up	
Work	Return to work, modified duties, hours
Social life	Satisfaction with social relationships
Health related quality of life	Likert scale for overall health, Global Perceived Effect (GPE) [25], EQ5D3L [23] and SF12 [24].
Compensation	Claim made, claim type, claim acceptance, fault/blame, legal representation, disputes, medico-legal assessments, claims costs, satisfaction with claims process, previous claim history.
Disability and functioning	History of any disability, World Health Organisation Disability Assessment Schedule II (WHODAS II) [28].
Psychological factors	Depression, Anxiety and Stress Scale (DASS21) [29] and Impact of Events Scale (IES-R) [30], expectation to recover using OMPQ.
Pain	Numeric rating scale (NRS) [26]

*In addition circumstances of crash will also recorded as open verbatim at 6 months.

### Baseline and follow-up data and study endpoints

Data from multiple domains will be recorded at baseline and the follow-up interviews as reported in Table 2. Standardised instruments are used that assess the relevant domains based on previous research.

The primary outcomes are health related quality of life, disability and functioning at 6, 12 and 24 months after injury in motor vehicle crash. The secondary outcomes are pain status, psychological health, return and modification to work status, social outcomes, role of compensation and health care utilisation.

### Data linkage

Pre-injury health status is recognised as a confounder in studies of health outcomes after motor vehicle crashes. It can be measured by recall from the injured person but this may be biased. To avoid this, participant consent will be obtained to access Medicare records for the 12 month period prior to the crash date and for two years after the crash. These data will be linked with the data collected directly from participants. The details of variables that will be collected from Medicare records are listed in Table 3. Previously a systematic review has reported consent rate to range between 39%-97% for data linkage [20]. It was observed that in general, individuals tend to consent to the use of their data for record linkage, with exceptions in specific populations or minorities. We anticipate a consent rate of approximately 80% for the study.

**Table 3:** Data collected through Medicare and Pharmaceutical Benefit scheme

**Variables from medicare data**	**Description**
Date of service	Date on which the provider performed the service
Date of processing	Date on which Medicare processed the payment of a claim for the service
Medicare benefits Item description	Describes the service provided by the provider as per Medicare Benefits Schedule
Medicare item number	A number that identifies the service provided by the provider as per Medicare Benefits Schedule
Provider charge	The amount the provider charged for the service
Schedule fee	The fee listed in the Medicare Benefits Schedule
Benefit paid	The Medicare benefit paid to the claimant
Patient out of pocket	The amount the patient is out of pocket i.e. provider charge minus benefit paid Bill
Type	Method by which the Medicare benefit was claimed i.e. cash, bulk bill, cheque to claimant, cheque to provider via claimant, PCE (Easy claim patient claim), simplified bill and EFT
Scrambled ordering provider number	A scrambled provider number identifying the doctor who referred the service scrambled
Rendering provider number	A scrambled provider number identifying the doctor who provided the service
Date of referral	The date of referral or request for a service by a provider
Rendering provider postcode	Postcode of servicing provider’s practice location
Ordering provider postcode	Postcode of referring provider's practice location
Hospital indicator	An indicator of whether the service was performed in hospital
Provider speciality	Speciality of the provider as at time of service
Item category	From the hierarchical system of the Medicare Benefits Schedule
**Variables from Pharmaceutical Benefits Scheme (PBS) claims data**	
Date of supply	The date on which the PBS item was supplied
Date of prescribing	The date on which the prescription was written PBS
Item code	Number which indicates item prescribed as per Schedule of
Pharmaceutical benefits item description	Item name description as it appears in the Schedule of PBS
Pharmaceutical benefits patient category	Refers to the patient's concessional status at the time of supply of the benefit of the item
Patient contribution	The contribution actually paid by the patient
Net benefit	Benefit that Medicare paid to the Pharmacy
Scrambled prescriber number	A scrambled prescriber number identifying the doctor who prescribed the PBS item
Pharmacy postcode	Postcode of Pharmacy where the prescription was dispensed
Form category	Description of script type, e.g. OR: Original, RE: Repeat, DS, Deferred Script, AU: Authority, AR: Authority Repeat
Code	Code allocated by the WHO Collaborating Centre for Drug Statistics
ATC name	According to the Anatomical Therapeutic Chemical (ATC) classification system
Prescriber derived major speciality	Speciality of prescribing doctor

### Study size

Allowing for 30% loss to follow-up a total of 2400 participants will be recruited at baseline. It is anticipated that a sample size of 1,500 is required at 24 months follow-up for linear and logistic regression statistical analyses. Allowing for a 25-30% refusal rate, nearly 3200 participants will be screened for the study. The sample size calculations are for 90% power, to detect independent effects of about 5% of the size of the variable in multiple linear regression analysis. These calculations are based on experience from the pilot study [31].

### Data protection

All screening data will be entered on a secure platform, the Research Electronic Data Capture (REDCap) which is maintained by the Information and Communication Department, University of Sydney. Additionally, data from REDCap will be stored on a password protected Excel database which will be secured on an additional password protected USB stick stored in a lockable cabinet. Cloudstor, a secure large file transfer system will be used for Internet data transfer among the research teams at University of Sydney and University of Queensland.

For Medicare and Pharmaceutical Benefit Scheme data linkage, four researchers of the team have been given access to the data. A protocol developed by the Australian Institute for Health and Welfare, for linking two or more data sets held by the institute will be complied with, for data linkage [31].

### Bias

We aim to minimise sampling bias by enrolling an inception cohort within 28 days of the crash event. Recruitment of participants from a range of different sources will result in a heterogeneous cohort of people injured in road crashes, including a range of injury severity. Participants will be broadly representative of the injured population of NSW in terms of injury severity and type, and demographic details. Recruitment utilising data police crash reports will allow recruitment of people injured in road crashes from rural and remote areas of NSW.

Measurement bias will be minimised by using pooled and de-identified data analysed by study investigators and staff who have not participated in recruitment or follow-up of individual participants. The study uses measurement scales and outcome instruments that have been previously shown to be valid and reliable, reducing measurement bias in the study. Interviews are conducted by trained staff using CADI.

Bias due to selective loss to follow-up will be minimised by using strategies such as, flexible time for calling, computer aided telephone interviews, engaging with participants via regular newsletters, and by recording multiple telephone numbers for contact so as to ensure high follow-up rates. Loss to follow up interview at any time point will not preclude an attempt to follow-up for subsequent interview. The Prospective Outcomes of Injury Study (POIS) study from New Zealand has reported 38% of those who did not participate at 12 months follow up interview participated at 24 months [32].

In the analysis phase, appropriate strategies will be used to assess potential confounding factors. While every attempt will be made to ensure complete data collection for all study participants, missing data are inevitable. If need be, the use of multiple imputation methods for missing data will be used to maximise available data. In multiple imputations, missing values for any variable are predicted using existing values from other variables. Multiple imputation accounts for missing data by restoring not only the natural variability in the missing data, but also by incorporating the uncertainty caused by estimating missing data [33]. The performance of multiple imputation in a variety of missing data situations has been well-studied and it has been shown to perform favourably [34,35].

### Data analysis

Summary statistics will be used to describe the profile of participants in the study at each time point. Frequencies and percentages, means and standard deviations, and medians and interquartile ranges, will be used to summarise key predictors and outcomes, depending on the distribution of the data collected for these variables. Key participant groups (for example, participants lost to follow-up vs. not lost to follow-up, compensable vs. non-compensable) will be compared using chi-square tests for categorical characteristics, and either independent t-tests or Mann–Whitney U-tests for continuous variables depending on the distributional characteristics of particular variables.

To analyse change in outcome over time, and to explore predictors of outcome, multilevel mixed effects regression models will be fitted with a random effect for patient to allow for excess correlation in outcomes measured repeatedly from the same participant. Mixed effects modelling is warranted due to the longitudinal nature of the study. Logistic regression will be used for binary outcomes and linear regression for continuous outcomes. Univariate and bivariate models will be performed to explore the association between individual predictors and each outcome. To establish whether the rate of change in the outcome differs across participant sub-groups (for example, compensable vs. non-compensable), an interaction term between each variable and follow up time point will be included in the regression model. Interaction terms between various predictors will be examined in order to identify potential effect modification. Multivariate mixed effects models will be used to identify important predictors of outcome, adjusted for potential confounders, and including key a priori interactions. Odds ratios, adjusted odds ratios, and 95% confidence intervals will be reported for logistic models, while coefficients and 95% confidence intervals will be reported for linear models.

### Ethics

The study protocol was approved by the Sydney Local Health District Ethics Committee; reference number HREC/13/CRGH/67. Site Specific Approvals were sought at each hospital site. The study has also been approved by Human Ethics Committee at University of Queensland and University of Sydney. For Medicare and Pharmaceutical Benefit Scheme data linkage ethical approval was received by Department of Ethics, Department of Health and Aging, Department of Health Services and the Ethics committee at Australian Institute of Health and Welfare.

The study was prospectively registered with Australia New Zealand Clinical trial registry, the trial identification number is ACTRN12613000889752.

## Discussion

The primary focus of this study is to examine associations and predictors for a range of outcomes, requiring recruitment of a study population that exhibits heterogeneity in the exposures of interest at baseline. It is hypothesised that people with certain pre-injury characteristics, such as poor pre-injury health, poorer social support, socio-economic factors including employment, less job satisfaction and immediate post-injury factors, injury severity, including higher pain and poorer mood, will have less favourable health and social outcomes and are more likely to seek compensation. Also, people dissatisfied with the claim process, outcome, delayed liability determination or denied liability, perceived injustice (blame others, not at fault) and entitlement (to financial compensation) will have less favourable health and social outcomes, adjusting for disability, pain and injury severity, and claim duration. It is also postulated that people with claims a greater number of, and higher cost, and would be less likely to receive evidence-based treatments from health care providers.

Our study protocol ensures heterogeneity through recruitment from a range of settings. This approach will allow recruitment of participants with a wide range of demographic, pre-injury, injury severity, compensable status and fault status factors. The anticipated high participation rates will allow recruitment of a generalizable sample which is broadly representative of the NSW injured population. The results are anticipated to make a major contribution for evidence based reforms in the Compulsory Third Party (CTP) scheme in NSW and future research.

*Expected implications for future research:* The data on predictor variables will help identify high risk groups and would lead to future research for targeted interventions. Identifying the factors influencing health and social outcomes is likely to lead to future research on modifiable factors like health care utilization for better outcomes in the population after a crash.

*Expected implications for policy:* It is hypothesised that compensation status is associated with health outcomes, health care utilization, return to work and legal representation. We hope that the results from the study will provide robust evidence for policy initiatives addressing how compensation factors could improve health and social outcomes and scheme efficiency and/or cost effectiveness of the NSW CTP scheme.

## Abbreviations

AIS: Abbreviated injury score; BMI: Body mass index; CTP: Compulsory third party insurance; DASS: Depression, Anxiety and Stress Scale; ESPARR: Rhône Area Road Crash Victim Follow-up Study; EQ-Qol: Epistaxis-specific quality-of-life questionnaire; GPE: Global perceived effect; IES-R: Impact of Event Scale- revised; MAA: Motor Accident Authority; MCS: Mental component score; NRS: Numeric rating scale; NSW: New South Wales; OMPQ: Orebro Musculoskeletal Pain Question; PCS: Physical component score; PCS: Pain catastrophising scale; POIS: Prospective outcomes of injury study; PRSS: Pain-related self-statement scale; SF: Short-form health survey; TAC: Transport Accident Commission; WHODASS II: World Health Organisation Disability Assessment Schedule II.

## Competing interests

The authors declare that they have no competing interests.

## Authors’ contributions

JJ drafted the manuscript, coordinates the study and contributed to the conception of the study. FB, SD, RI, CGM, JK, AC, MN, SB, SW and BG contributed to the conception of the study. IC designed the study, drafted the grant proposal, contributes to the coordination of the study and is the principal investigator of the study. All authors critically revised the manuscript and agreed to the final version of the manuscript.

## Pre-publication history

The pre-publication history for this paper can be accessed here:

http://www.biomedcentral.com/1471-2458/14/199/prepub
